# Handgrip Strength as a Diagnostic Tool in Work-Related Upper Extremity Musculoskeletal Disorders in Women

**DOI:** 10.1100/tsw.2004.12

**Published:** 2004-03-03

**Authors:** Deborah Alperovitch-Najenson, Eli Carmeli, Raymond Coleman, Haim Ring

**Affiliations:** ^1^ Department of Physiotherapy, Sackler Faculty of Medicine, Stanley Steyer School of Health Professions, Tel Aviv University, Ramat Aviv 69978, Israel; ^2^ Department of Anatomy and Cell Biology, Bruce Rappaport Faculty of Medicine, Technion-Israel Institute of Technology, Haifa, Israel; ^3^ Loewenstein Hospital Rehabilitation Center and Sackler Faculty of Medicine, Tel Aviv University, Ramat Aviv 69978, Israel

**Keywords:** handgrip strength, musculoskeletal disorders, women, factory workers, public health, Israel

## Abstract

The aim of this study was to determine if handgrip strength might be used as a diagnostic tool in musculoskeletal disorders of the upper extremities in women working in an industrial environment. The setting was an electronic factory with four groups of women (n = 101) in a factory assembling electronic components. Handgrip strength was measured using a Jamar® hydraulic hand dynamometer. The study investigated grip strength in managers-engineers, cable wiring, circuit board assembly, integrated circuits women at 90? elbow flexion and 180? elbow extension. Women seeking or receiving medical care for musculoskeletal disorders of the upper extremities or neck showed significant declines (*p* < 0.01) in handgrip strength and these also related to the type of work and the level of perceived physical exertion. Women in the managerial-engineering group showed fewer musculoskeletal disorders of the upper extremity compared with the other groups and also had significantly stronger handgrip. Our findings encourage us to recommend hand dynamometer testing as a useful diagnostic tool to determine loss of handgrip strength.

## INTRODUCTION

Work-related musculoskeletal disorders (WMSDs) of the upper extremities are common causes of pain and functional decline and can lead to significant distress and disability[1,2]. Identifying the factors associated with reduced upper extremity function may lead to the development of more effective interventions. Various risk factors may be involved including biomechanical and environmental conditions such as physical work load, unfavorable body posture, vibration[3,4,5,6,7,8], and psychosocial factors such as time pressure and repetitive or monotonous tasks[9,10,11]. Musculoskeletal problems are commonly described as cumulative traumatic disorders, repetitive strain injuries, repetitive use injuries, occupational overuse disorders, and repetitive motion disorders[11]. We have elected to use the term "work-related musculoskeletal disorders" as recommended by Morse et al.[12].

The initial episodes of a WMSD typically occur within the first years of work and are commonly diagnosed as a result of pain or functional decline. The purpose of our study was to test handgrip strength in several categories of women employed in an electronics factory to try and identify if there is a relationship between handgrip strength, work patterns, and acquired musculoskeletal disorders of the upper extremity. We attempted to identify specific work conditions and sociodemographic parameters that possibly may contribute to the occurrence and severity of the musculoskeletal disorders. Our postulate was that women in specific work environments complaining of upper extremity pain may also demonstrate decreased handgrip power, and that this can possibly be used as a diagnostic measure of functional decline. An improved understanding and awareness of potential environmental causes of work-related musculoskeletal problems may contribute to improvements in work organization and design of the workplace. Improved ergonomics could result in reduced stress levels and lower incidence of work-related musculoskeletal complaints[13,14].

## METHODS

One hundred and one women were investigated in a cross-sectional study (age range 23–63, mean 41.4 ± 9.6). The women were engaged in mainly sedentary work in four different departments of the same electronics factory (managers-engineers, n = 29; cable wiring, n = 24; board-assembly [facing the table and working while sitting], n = 20; integrated circuits [quality control of products after machine manufacture], n = 28). The study was done in Israel, in a naturalistic factory setting near Tel Aviv. The four departments involve different repetitive use of the upper extremities.

Two basic categories were identified in the study: an "asymptomatic group" (60 women, who had not sought medical care during the previous 5 years for neck, shoulder, or upper extremity pain), and a "symptomatic group" (41 women, who sought medical care over a similar period of time). Pregnant women or women suffering from neurological or orthopedic diseases, both chronic and acute, that might disturb upper extremity function, were excluded from the study.

An occupational physician undertook an initial interview to assess general health and record medical histories and socioergonomic data (age, body mass index, educational status, seniority, occupation type, and perceived physical exertion of neck and upper extremities at work). Women with early onset of menopause were excluded from the study. Information about the onset of the musculoskeletal symptoms, treatment and pain resolution of the "symptomatic group" were taken and analyzed yet found irrelevant to be presented here. Perceived physical exertion was rated according to Borg's new Rating of Perceived Exertion on a scale of 1–10, where 0 = nothing at all, 5 = strong, and 10 = very strong[15]. The perceived exertion rating was collected while subjects were working yet in a quite and separated room.

The functional testing of handgrip strength was measured using a standard Jamar® hydraulic hand dynamometer (G100, NexGen Ergonomoc, Montreal, Canada). This diagnostic tool was found sensitive and specific for a working population[16]. The test was administered with the subject standing with the upper extremity in two different positions: with the elbow attached to the body at 90º flexion, and also with the upper extremity extended straight forward. In both cases, the wrist was in slight extension, in a position determined by the subject as comfortable[17]. The second position, with the elbow straight, was selected as the functional position, involving more of the upper limb. The subject was asked to grip the dynamometer, press as hard as possible, and relax immediately (submaximal force). Each test was carried out on both upper extremities intermittently, and repeated three times. The tests were performed without pain. To evaluate grip strength using the Jamar® hand dynamometer, a second or third grade of the five grades available of hand opening dependent on the best average result was used as recommended[18]. The average of three repetitions was calculated. The examiner was blinded as to which group the subjects under evaluation were from, and all tests were done not on a workday thus to prevent fatigue. A repetitive calibration of the dynamometer was done every 15 measurements.

## Statistic Analyses

A comparative analysis of the distribution of the study parameters of the symptomatic and asymptomatic groups is presented. Pearson product-moment correlation coefficient and Spearman rank correlation coefficient were applied for testing the correlation between the study parameters examined. ANOVA test was used to compare between the study groups according to occupation. The age discrepancy between the two groups was control, thus in term of grip strength comparisons the two groups were well matched. Chi Square test was used to compare between the study groups according to perceived levels of physical exertion. T test was used to compare between averages of the study parameters between the study groups. Multiple logistic regression was applied to test the odds ratio of the research variables (age, body mass index, years of study, occupation type, perceived physical exertion) as factors influencing the symptomatic and the asymptomatic groups. An adjustment was made to compensate for age differences between the study groups, thus age appeared to be accounted for by the study design.

## RESULTS

In the socioergonomic comparison between the symptomatic and asymptomatic groups using the variables of age, body mass index, years of study, seniority, occupation type, and perceived physical exertion of neck and upper extremities at work, several significant differences were found. These included: age (45 ± 4.6 and 38 ± 3.8 years, respectively, $p = 0.001$), seniority (17 and 13 years, respectively, $p = 0.032$), occupation type, and perceived physical exertion of neck and upper extremities.

A significantly higher percentage (72%) of women of the managerial-engineering category belonged to the asymptomatic group ($p = 0.048$), whereas only 28% belonged to the symptomatic group. The percentage of symptomatic women in this group was considerably lower than that of the three other occupation groups. The cable wiring and board assembly groups showed almost identical percentages in the symptomatic and asymptomatic groups. In the group of women working on the integrated circuits, 40% were symptomatic and 60% asymptomatic.

Distribution of subjects according to perceived physical exertion of the neck and upper limbs showed that slightly more women in the symptomatic group than in the asymptomatic group complained of high perceived physical exertion (Fig. 1); 26% of participants (n = 12) with low perceived physical exertion belonged to the symptomatic group, whereas 74% (n = 34) belonged to the symptomatic group.

Maximal grip strength was compared between the symptomatic and the asymptomatic groups (see Table 1). Significant differences were found between the two research groups with regard to the right hand with the elbow straight ($p = 0.02$). All but nine participants (9%) were right-handed, no differences between sides were noticed. Namely, the mean grip strength of the right hand was greater in the asymptomatic group compared with the symptomatic group. Examination with the elbow flexed did not show significant differences between the research groups with regard to either the right or the left hands. A significant difference was found in the comparison between the grip strength differences of the right and left hands, both with the elbow flexed and extended ($p = 0.03$ and $p = 0.008$, respectively). Namely, the average of differences between grip strength of both hands was greater in the asymptomatic group compared with the symptomatic group.

The relationship between individuals belonging to the symptomatic group and socioergonomic variables (age, seniority, and perceived physical exertion) was examined. The correlation was found to be 0.35, 0.20, and 0.27, respectively ($p < 0.05$ for all results).

Odds ratios of sociodemographic variables in the symptomatic group compared with the asymptomatic group are shown in Table 2. The three more physical occupations (cable wiring, board assembly, and integrated circuits assembly) were compared to the fourth occupation (managers- engineers).

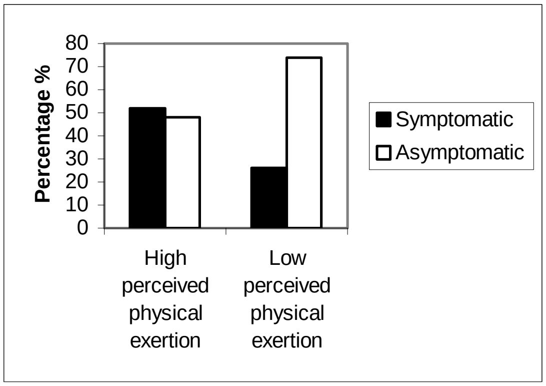

**FIGURE 1.** Distribution of participants according to perceived physical exertion of the neck and upper limbs and research groups (symptomatic and asymptomatic).

**TABLE 1**
**Maximal Grip Strength of Second and Third Grade Hand Opening Out of Five**

| Hand and Elbow | Asymptomatic Group (n =60) | Symptomatic Group (n = 41) | *t*-test | *p* |
|---|---|---|---|---|
| Right hand elbow flexed 90º | 25.21 ± 5.12 | 23.76 ± 5.29 | 1.36 | NS |
| Left hand elbow flexed 90º | 23.11 ± 4.51 | 22.92 ± 5.54 | 0.18 | NS |
| Difference | 2.09 ± 2.38 | 0.83 ± 3.64 | 2.09 | 0.03 |
| Right hand elbow straight | 25.27 ± 5.40 | 22.35 ± 6.49 | 2.37 | 0.02 |
| Left hand elbow straight | 22.90 ± 4.88 | 21.63 ± 6.31 | 1.13 | NS |
| Difference | 2.37 ± 2.17 | 0.72 ± 3.94 | 2.69 | 0.008 |

Mean values ± SD in kg. NS, not significant.

**TABLE 2**
**Odds Ratio of Sociodemographic Variables in the Symptomatic Group Compared with the Asymptomatic Group**

| Variable | Odds Ratio | Confidence Interval | *p* |
|---|---|---|---|
| Age | 1.09 | 1.01–1.17 | 0.01 |
| Perceived physical exertion | 3.31 | 0.97–11.28 | 0.05 |
| Years of study | 1.02 | 0.81–1.28 | 0.82 |
| Body Mass Index | 1.02 | 0.98–1.06 | 0.27 |
| Cable wiring* | 0.46 | 0.11–1.98 | 0.30 |
| Board assembly* | 0.27 | 0.05–1.39 | 0.43 |
| Integrated circuit assembly* | 0.57 | 0.12–2.69 | 0.48 |

*The three more physical occupations in the table (cable wiring, board assembly, and integrated circuit assembly) were compared to the fourth occupation (managers-engineers).

Odds ratio being in an ergonomic environment of higher perceived physical exertion is more significant among the symptomatic group subjects (OR = 3.31).

Odds ratio of sociodemographic variables in the higher perceived physical exertion group compared with the lower perceived physical exertion group is shown in Table 3. Odds ratio of working among the cable wiring occupation and the board assembly occupation is 61.49 and 20.76, respectively.

**TABLE 3**
**Odds Ratio of Sociodemographic Variables in the High Perceived Physical Exertion Group Compared with the Low Perceived Physical Exertion Group**

| Variable | Odds Ratio | Confidence Interval | *p* |
|---|---|---|---|
| Age | 0.99 | 0.91–1.08 | 0.91 |
| Symptomatic group | 3.18 | 0.97–10.39 | 0.05 |
| Years of study | 0.77 | 0.56–1.04 | 0.09 |
| Body Mass Index | 1.02 | 0.97–1.07 | 0.37 |
| Cable wiring | 61.49 | 6.45–58.62 | 0.0003 |
| Board assembly | 20.76 | 2.77–15.53 | 0.003 |
| Integrated circuit assembly | 2.68 | 0.43–16.40 | 0.35 |

## DISCUSSION

Our study was performed in a clinical environment (not on the factory floor) and reflects the clinical conditions resulting from musculoskeletal disorders of the upper extremities induced by repetitive work activities and stress. This study of women working in industry showed that the group that sought or received medical care for musculoskeletal disorders of the upper extremities or neck (symptomatic group) showed significant declines in handgrip power overall compared with the asymptomatic group. Hand dynamometers are used traditionally to measure grip force even in the absence of any upper limb abnormality. The dynamometric data were tested to assess WMSD of the upper extremities that cause pain and functional decline. High WMSD prevalence when tested in two different positions of the arms was shown to depend to a large degree on the occupation type of the participants in the study. Postural load in the workplace plays an important role in the development of musculoskeletal disorders[3,4] and our study may point for the need to improve work conditions and reduce postural stress in these groups of workers.

The effect of arm position on grip strength was tested to evaluate the integrated performances of the neck and upper limb muscles during different tasks. Most subjects reported they felt the same with arm extended as with flexed elbow, however the high interclass coefficient values did not support this. Grip strength was presumably decreased in the symptomatic group especially with the elbow positioned in extension. Certain works require the using of two hands separately or even simultaneously. Therefore, we evaluated left and right hand, and in the asymptomatic group a higher muscle strength was observed in the dominant side.

Our findings indicated that there is a difference in grip strength between arm positions with a significant reduction in handgrip in the right hand with the arm straight and not with flexed elbow. Su et al.[19] found that highest grip strength score was obtained when the shoulder was flexed at 180˚ with the elbow fully extended, and it decreased as the shoulder was positioned in lesser degrees of flexion. The lower score was found at 0˚ of shoulder flexion with elbow flexed at 90˚. In our study, we differentiated between two groups and performed statistical analysis separately. However there was a reduction in grip strength in the 90º elbow flexed position in the symptomatic group. Few researchers[19,20] using electromyography suggested that the most two likely explanations of this could be: (1) that for both elbow positions different amounts of synergistic activities occur in the back and shoulder muscles, and (2) alterations in proprioception and contractions usually follow chronic musculoskeletal injuries thus affecting handgrip, especially in the shoulder-flexed position. In addition, as reported before[21], the mean grip strength in the dominant hand was greater in the asymptomatic group compared with the symptomatic group. Higher strength levels depend on increased discharge frequency in each motor unit, and this function may be compromised in WMSD associated with chronic muscle injury[22]. WMSD may also be associated with microtrauma of the myofilaments resulting in reduced muscle strength[18]. However, it is difficult to conclude whether pain leads to decrease in muscle strength or decrease in muscle strength leads to pain. Several mechanisms may explain the decrement of WMSD: (1) biomechanical stress such as repetitive movements; (2) biochemical or physiological stress such as soft tissue inflammation and accumulation of agents-related pain such as cytokines, free radicals, and blood supply interference[23], as well as psychological or social stress[24].

Previous studies have established relationships between hand strength and variables such as age, years of study, body mass index, and seniority[25]. Our results of the mean strength values with 90º and 180º elbow range of motion were similar to those reported by Mathiowetz et al.[25] The question whether the above variables are capable of affecting the prehensile strength in both normal and WMSD individuals has been investigated. Sande et al.[18] indicated that only weight was a factor with a significant influence on strength.

Our findings encourage us to recommend hand dynamometer testing as a useful diagnostic tool to determine loss of handgrip strength, which may indicate development of musculoskeletal disorders of the upper extremities. Owing to the low cost, simplicity, speed, and pain-free nature of the test to determine handgrip strength, regular testing and evaluation can provide indications of work conditions that have a direct impact on musculoskeletal disorders. The tests may indicate the need for individual adaptation to specific work conditions and behavior, including education and preventive training. Improved ergonomic work conditions on the factory floor can reduce WMSD and promote overall improved health. Systematic handgrip strength testing can provide a simple and useful tool in occupational health research and in prevention/intervention strategies.

## ACKNOWLEDGMENTS

The authors wish to express their gratitude to Anne and Eli Shapira Charitable Foundations, Portland, Oregon, that supported this study.

## BIOSKETCHES

**Deborah Alperovitch-Najenson, MoccH, PT**, is affiliated with the Sackler Faculty of Medicine, Stanley Steyer School of Health Professions, Department of Physical Therapy, Tel Aviv University, Israel.

**Eli Carmeli, PT, PhD**, is currently working in a full-time tenure tract position at the Physical Therapy Department, Sackler Faculty of Medicine, Tel Aviv University. He is investigating the aging process both on the cellular and clinical level. E-mail: elie@post.tau.ac.il

**Raymond Coleman, PhD,** is Associate Professor at the Department of Anatomy and Cell Biology, Bruce Rappaport Faculty of Medicine, Technion-Israel Institute of Technology, Haifa, Israel with research interest in histochemistry, microscopy, image-analysis, and aging. E-mail: coleman@tx.technion.ac.il

**Haim Ring, PhD, MD**, is affiliated with the Loewenstein Hospital Rehabilitation Center and Sackler Faculty of Medicine, Tel Aviv University, Israel.
